# Bioinformatic Approach to Unveil Key Differentially Expressed Proteins in Human Sperm After Slow and Rapid Cryopreservation

**DOI:** 10.3389/fcell.2021.759354

**Published:** 2022-01-25

**Authors:** Pedro O. Corda, Joana Vieira Silva, Sara C. Pereira, Alberto Barros, Marco G. Alves, Margarida Fardilha

**Affiliations:** ^1^ Department of Medical Sciences, Institute of Biomedicine (iBiMED), University of Aveiro, Aveiro, Portugal; ^2^ Department of Chemistry, QOPNA and LAQV, University of Aveiro, Aveiro, Portugal; ^3^ Clinical and Experimental Endocrinology, Department of Anatomy and Unit for Multidisciplinary Research in Biomedicine, Institute of Biomedical Sciences Abel Salazar, University of Porto, Porto, Portugal; ^4^ Centre for Reproductive Genetics A. Barros, Porto, Portugal; ^5^ Department of Genetics, Faculty of Medicine, University of Porto, Porto, Portugal

**Keywords:** sperm cryopreservation, proteomic analysis, bioinformatic tools, ARSA, biomarker candidates

## Abstract

Currently, two conventional freezing techniques are used in sperm cryopreservation: slow freezing (SF) and rapid freezing (RF). Despite the protocolar improvements, cryopreservation still induces significant alterations in spermatozoon that are poorly understood. Here, available proteomic data from human cryopreserved sperm was analyzed through bioinformatic tools to unveil key differentially expressed proteins (DEPs) that can be used as modulation targets or quality markers. From the included proteomic studies, 160 and 555 DEPs were collected for SF and RF groups, respectively. For each group, an integrative network was constructed using gene ontology and protein-protein interaction data to identify key DEPs. Among them, arylsulfatase A (ARSA) was highlighted in both freezing networks, and low ARSA levels have been associated with poor-sperm quality. Thus, ARSA was selected for further experimental investigation and its levels were assessed in cryopreserved samples by western blot. ARSA levels were significantly decreased in RF and SF samples (∼31.97 and ∼39.28%, respectively). The bioinformatic analysis also revealed that the DEPs were strongly associated with proteasomal and translation pathways. The purposed bioinformatic approach allowed the identification of potential key DEPs in freeze-thawed human spermatozoa. ARSA has the potential to be used as a marker to assess sperm quality after cryopreservation.

## Introduction

Sperm cryopreservation is a key procedure in reproductive medicine worldwide. This technique has been used extensively to preserve male fertility in situations that can lead to testicular dysfunction and ejaculatory failures such as some pathological scenarios (malignant cancers and severe spinal cord injuries), corrective surgeries, or chemo-/radio-therapies (Di Santo et al., 2012; Tournaye et al., 2014; Tomlinson et al., 2015). Also, sperm cryopreservation has proved to be useful in several male infertile conditions, such as azoospermia and oligozoospermia (Di Santo et al., 2012). Nowadays, two conventional freezing techniques are used in sperm cryopreservation: slow freezing (SF) and rapid freezing (RF) (World Health Organization, 2010). In SF, the sperm sample is progressively cooled in a period of 2–4 h until it reaches -100°C (World Health Organization, 2010; Di Santo et al., 2012). In contrast, sperm samples frozen through a RF protocol are exposed to nitrogen vapors (-80°C) during 10–15 min before being placed in liquid nitrogen (-196°C) (World Health Organization, 2010; Di Santo et al., 2012). Although the efforts made in the last decades, the current cryopreservation protocols are still far from ideal inducing significant deleterious structural and molecular alterations in spermatozoa (Hezavehei et al., 2018; Rienzi et al., 2018; Kumar et al., 2019).

The primary cause of cryoinjury is water phase changes in low temperatures that lead to ice crystal formation (intra- and/or extracellular). In RF, water movement is impaired which leads to intracellular ice crystal formation (Said et al., 2010; Di Santo et al., 2012). On the other hand, when the cooling rate is too slow, there is water efflux to the extracellular environment which contributes to the formation of extracellular ice crystals and intracellular osmotic pressure increase (Said et al., 2010; Di Santo et al., 2012). In both situations, there is cell membranes disruption, organelle damage and, consequently, cell viability loss (Said et al., 2010; Di Santo et al., 2012; Jang et al., 2017; Hezavehei et al., 2018). Moreover, the cooling temperatures induce changes in the phase transition of lipid layers and membrane proteins’ function (Quinn, 1985; Talaei et al., 2010). The cryoinjuries are not limited to the freezing steps and may occur during the thawing process due to ice melting or recrystallization (Verheyen et al., 1993). Sperm cryopreservation also induces changes in mitochondrial membrane fluidity (O’Connell et al., 2002). The mitochondrial membrane potential is compromised, and therefore the ATP synthesis is reduced. This event may explain the motility reduction of thawed spermatozoa since mitochondrial ATP is one of the main sources of energy in sperm (O’Connell et al., 2002; Amaral et al., 2013). Additionally, mitochondrial membrane changes induce ROS release and promote spermatozoon oxidative damages such as membranes impairment and destabilization of axonemal structures (Saleh and Agarwal, 2002; Talaei et al., 2010). The activity of the antioxidant enzymes is decreased during cryopreservation which contributes to an increase in ROS damage (Lasso et al., 1994; Banihani and Alawneh, 2019). High ROS concentrations and the failure of antioxidant activity lead to the activation of sperm apoptotic pathways and, consequently, the release of apoptosis-inducing factors (Martin et al., 2007; Said et al., 2010; Karabulut et al., 2018). Together, those changes significantly reduce sperm motility, viability, acrosome integrity, and fertilization ability of freeze-thawed spermatozoa (Kumar et al., 2019). Despite the known consequences of sperm cryopreservation, the molecular mechanisms responsible remain largely uncharacterized.

The emergence of proteomic techniques brought a new perspective on the study of sperm physiology, allowing the evaluation of the protein profile in certain physiological/pathological contexts and the molecular understanding of complex processes such as motility (Xu et al., 2012b; Cao et al., 2018; De Rose et al., 2018). To date, few proteomic studies have been carried out to understand the proteomic changes underlying sperm cryopreservation. Most of them were performed in animal models such as bovine and pig (Parrilla et al., 2019; Ryu et al., 2019). In 2014, Wang and colleagues published the first proteomic study using cryopreserved human spermatozoa (Wang et al., 2014). They compared the proteomic changes between freeze-thawed spermatozoa and fresh controls from normozoospermic donors. Twenty-seven differentially expressed proteins (DEPs) were identified in the cryopreserved samples, being most of them associated with sperm motility, acrosome integrity, capacitation, viability, and mitochondrial activity (Wang et al., 2014).

The present work aims to identify key DEPs in human spermatozoa after SF and RF that can be used as modulation targets or quality biomarkers. For that purpose, proteomic data was collected from the available studies that compared freeze-thawed human spermatozoa with fresh controls, and, through a bioinformatic workflow, a set of proteins that seem to be relevant among the DEPs was isolated. From those proteins, arylsulfatase A (ARSA)—a sperm membrane protein—was investigated as a potential marker for quality assessment in cryopreserved sperm samples.

## Materials and Methods

### Search Strategy

An exhaustive literature search was conducted in the PubMed database to identify proteomic studies that compare changes in cryopreserved vs fresh controls human spermatozoa. Only studies published in English (or at least with English abstract) until May 18th, 2020, were considered. From those, only the ones that meet the following inclusion criteria were selected: 1) use of ejaculated human samples from normozoospermic healthy donors; 2) use of fresh samples as control; 3) cryopreservation performed through slow or rapid freezing methods; 4) thawing process performed at temperatures equal or higher than 23°C; 5) protein extraction from whole spermatozoa in the sample; 6) quantitative proteomics; and 6) a false discovery rate (FDR) < 5% for protein identification.

### Collection of DEPs in Cryopreserved Human Spermatozoa

From each proteomic study selected, only DEPs (*p*-value <0.05) were collected. Considering the two conventional freezing methods, proteins were divided into two groups: SF and RF. Based on the fold-change ratio (comparison of protein levels between control and freeze-thawed samples in each study), proteins were also tagged as increased or decreased. To avoid redundancy, all DEPs were mapped in the UniProt database (downloaded on May 26th, 2020) and annotated using the UniProtKB/Swiss-Prot accession number. Then, the UniProt lists were cross-compared with human spermatozoon proteome (Santiago et al., 2019) through a Venn diagram analysis using the JVenn tool (Bardou et al., 2014). Only proteins with a reviewed status and previously reported in human spermatozoon were used for further analysis. An additional Venn diagram analysis was performed to identify common proteins among rapid and slow freezing groups.

### Gene Ontology and KEGG Pathways Enrichment Analyses of the DEPs

To gain insight into the molecular role of DEPs, a Gene Ontology (GO) enrichment analysis for biological process, molecular function, and the cellular compartment was performed for each group. This analysis was made through the ClueGO plugin (version 2.5.5) in Cytoscape (version 3.7.2) (Shannon et al., 2003; Trindade et al., 2018). Moreover, a KEGG pathway enrichment analysis was performed using the same plugin. The increased and decreased proteins of each group were uploaded in separated clusters to identify associated terms. The default settings were used in the SF group analysis. For the RF group, a more restrictive GO range, between 7 and 12, was used, and the GO term perfusion option was selected. Default settings were used in KEGG pathways analyses for both groups. Only GO and KEGG terms with a *p*-value <0.05 were considered.

### Integrative Protein-Protein Interaction Network Construction

To identify potential key DEPs in SF and RF groups, two independent integrative networks were built using protein-protein interaction (PPI) and enrichment analyses data. The String database (v11.0) was used to collect PPIs among the DEPs in slow and rapid freezing groups (Szklarczyk et al., 2019). Only experimental data with a high confidence score (0.700) were considered. For each freezing group, the 10 most significant biological processes, cellular compartments, and KEGG terms were included with the respectively associated proteins. Furthermore, common terms of both freezing groups were also used in the network construction. Network visualization was performed using Cytoscape. To identify the protein hubs of each network, the NetworkAnalyzer tool was used to compute the network topological properties (Shannon et al., 2003; Assenov et al., 2008). The top 10 proteins with the highest node degree were considered network hubs. For each protein hub, a search was conducted in DisGeNET (v7.0) and PubMed databases to identify an association between the protein and poor sperm quality or male (in)fertility.

### Ethics Statement

Human sperm samples were collected at the Centre for Reproductive Genetics Professor Alberto Barros after approval by the Joint Ethics Committee CHUP/ICBAS (2021/CE/P002 [P342/CETI/ICBAS]). All procedures were conducted following the ethical guidelines for human samples research. Donors signed an informed consent allowing the use of the samples for scientific purposes. The Centre for Reproductive Genetics A. Barros infertility clinic’s procedure is under the provisions of the National Medically Assisted Procreation Act (Law of 2017) and overseen by the National Council for Medically Assisted Procreation (CNPMA-2018).

### Human Sperm Samples

Semen samples were obtained from healthy male donors. Only sperm samples from normozoospermic donors (*n* = 15) were included in this study (mean age of 39.3 ± 5.20 years old). The semen samples were collected by masturbation into sterile containers, after an abstinence period of 2–7 days. Following complete liquefaction, basic semen analyses were performed according to World Health Organization (WHO)’s guidelines (World Health Organization, 2010). Then, each semen sample was divided equally into three aliquots: control, RF, and SF. For control samples, viability and motility were analyzed immediately.

### Cryopreservation and Thawing of the Semen Samples

Sperm cryopreservation was performed following the WHO guidelines (World Health Organization, 2010). The RF and SF semen samples were cryopreserved with Sperm Freezing Medium (Origio, Hovedstaden, Denmark) according to the manufacturer’s instructions. Briefly, Sperm Freezing Medium was added, slowly and dropwise, to each rapid and slow freezing semen sample (1:1, v/v), and then carefully mixed. The solution was maintained at room temperature (RT) for 10 min and subsequently transferred to cryovials. RF cryovials were placed at a -80°C refrigerator freezer for 30 min. SF cryovials were placed at a -20°C refrigerator freezer for 30 min and then transferred to a -80°C freezer for 30 min. In the end, SF and RF cryovials were immersed in liquid nitrogen (-196°C). After 8 days, the frozen samples were thawed by being placed in a pre-warmed water bath (23°C) for 15 min. The post-thawed parameters of motility and vitality were analyzed.

### Cell Viability Assay

Spermatozoa viability was evaluated through the eosin-nigrosine staining, as previously describe (Carrageta et al., 2020). Briefly, spermatozoa suspensions were mixed with an equal volume of 0.5% eosin-nigrosine stain and smeared onto a glass microscope slide; 200 spermatozoa were counted per replicate with the use of a light microscope. White spermatozoa were considered viable whereas pink-stained spermatozoa were considered non-viable.

### Motility Assay

Sperm total motility and progressive motility were assessed using a Makler counting chamber (Sefi Medical Instruments, Haifa, Israel), according to the manufacturer’s recommendations. A 10 µL of sperm suspension was placed in the center of the chamber and sperm motility was observed in 20 squares using an optical microscope. A total of 100 spermatozoa were counted per sample.

### Whole Sperm Lysates

Control and freeze-thawed semen samples were washed twice in sterile phosphate-buffered saline (PBS) by centrifugation (500x g, 5 min at 37°C) to remove the seminal plasma and cryoprotectant solution. Next, spermatozoa pellets were lysate in 1% sodium dodecyl sulfate (SDS; Acros Organics, Belgium) solution for 15 min on ice, followed by sonication (3 bursts, 5 s each with ice-cooling between bursts) (Naz, 1999). The lysates were centrifugated 16,000x g for 15 min at 4°C and the supernatant, corresponding to sperm soluble protein, was collected and stored at -20°C for subsequent use. For each sample, protein concentration was measured using bicinchoninic acid (BCA) assay (Pierce Biotechnology, Waltham, Massachusetts, United States) following the manufacturer’s instruction. The final absorbance was measured at 562 nm in a microplate reader (TECAN, Genius, Männedorf, Switzerland).

### Western Blot Analysis

To investigate the impact of cryopreservation in ARSA levels, sperm extracts corresponding to 7.5 µg of protein were resolved 10% SDS-polyacrylamide gel electrophoresis, and proteins were electro-transferred onto nitrocellulose membranes (Amersham, 0.45 µm). For loading control, membranes were stained with Ponceau S solution (Sigma Aldrich; 0.1% [w/v] in 5% acetic acid) for 15 min at RT with slow agitation. Membranes were washed with distilled water until protein bands were well defined and analyzed using densitometer GS-800. To remove the Ponceau S staining, the membrane was washed with 1x tris buffered saline containing 0.1% Tween 20 (1x TBST). Then, non-specific protein-binding sites on membranes were blocked for an hour with 5% bovine serum albumin (BSA)/TBST followed by incubation of the primary antibodies for 1 h at RT. Primary antibodies were used as follows: mouse anti-ARSA (Santa Cruz Biotechnology, Germany, catalog number: sc-365176) at 1:500; rabbit anti-β-tubulin (Proteintech Europe, UK, catalog number: 10094-1-AP) at 1:1000. After primary incubation, membranes were washed twice in 1x TBST for 10 min at RT and then incubated with appropriate secondary antibody: IRDye®680RD anti-rabbit (catalog number: 926–68,071) and IRDye®800CW anti-mouse (catalog number: 926–32,210). Both were diluted at the final concentration of 1:10,000 and purchased from LI-COR Biosciences (US). After the secondary incubation, membranes were washed three times for 10 min with 1x TBST and immunodetected using Odyssey Infrared Imaging System (LI-COR Biosciences, US). Ponceau and blot results were analyzed in the Image Studio Lite Version 5.2.5 Software (LI-COR Biosciences, Lincoln, Nebraska, United States). Bands’ intensity was normalized to Ponceau staining.

### Statistical Analysis

The statistical analysis was performed using GraphPad Prism (v8.0.1). Datasets were tested for normal distribution using the Shapiro-Wilk’s test. Since normality was not observed, the Mann-Whitney *U* test was used to compare vitality, motility, and protein levels in each freezing condition to the control group. Values of *p* < 0.05 were considered statistically significant. All experimental data are presented as mean ± standard deviation (SD). For ARSA levels, statistical power was assessed using the GPower (v3.1) tool, through the posthoc power analysis option for Wilcoxon-Mann-Whitney tests (Mayr et al., 2007).

## Results

### Description of the Selected Proteomic Studies

From the search conducted in the PubMed database, only five proteomic studies which used samples from normozoospermic healthy donors were identified (Wang et al., 2014; Bogle et al., 2017; Gholami et al., 2018; Fu et al., 2019; Li et al., 2019). From those, the Wang *et al.* (Wang et al., 2014) study was excluded since the thawed samples were washed through a Percoll gradient. The Gholami *et al.* (Gholami et al., 2018) study was also excluded because only the sperm tails were analyzed. Therefore, for the present analysis, only the three remaining studies (Bogle et al., 2017; Fu et al., 2019; Li et al., 2019), which fit the defined inclusion criteria, were considered eligible. In the study of Bogle *et al.* (Bogle et al., 2017), two methods of thawing were performed: 1) samples placed in a 23°C bath (15–20 s) and then placed on ice (∼0°C); or 2) samples placed and maintained in a 23°C bath. Thus, only the set of proteins identified in samples thawed by the second method were considered for subsequent analysis.

### Identification of the DEPs in Slow and Rapid Freezing Groups

**FIGURE 1:**
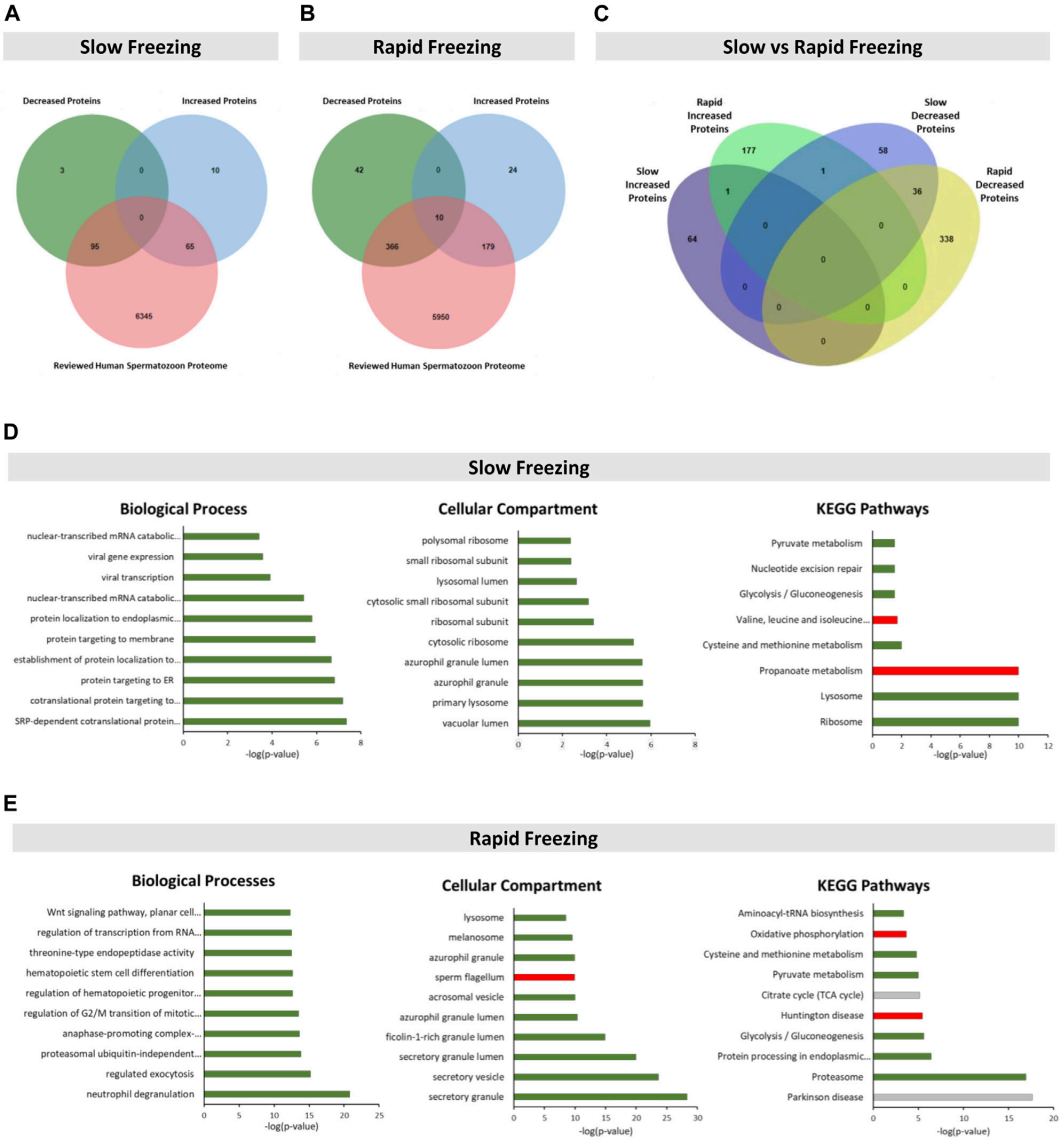
Identification and enrichment analysis of the DEPs in slow and rapid freezing groups. **(A,B)** Venn’s diagram illustrates the cross-comparison performed between DEPs of each freezing group and the reviewed human spermatozoon proteome. For the subsequent analyses, only DEPs reviewed and previously described in human spermatozoon were considered. In Venn’s diagram of the rapid freezing group **(B)**, the 10 DEPs that were described as increased and decreased were not considered. **(C)** Venn’s diagram shows the common DEPs between slow and rapid freezing groups **(D,E)** Top 10 significant terms for biological processes, cellular components, and KEGG pathways related with DEPs of slow and rapid freezing groups, respectively. Enrichment analyses were performed using the ClueGO plugin through Cytoscape. In each graph, the bar color represents a specific association as follows: green for decreased proteins; red for increased proteins; and grey for non-specific associations. The significant terms were identified by Bonferroni’s step-down corrected *p*-value (*p* < 0.01).

By merging data from the selected studies (Bogle et al., 2017; Fu et al., 2019; Li et al., 2019), 173 and 621 DEPs in sperm cryopreserved through slow and rapid freezing methods, respectively, were identified (Supplementary Table S1). In the SF group, 160 proteins had a reviewed status and were previously identified in human spermatozoon, of which 95 were decreased and 65 were increased (Figure 1A)**.** In the RF group, there were 555 human sperm-reviewed proteins (Figure 1B). From those, 366 were decreased, 179 proteins were increased, and 10 proteins were described as increased and decreased (blue highlight in Supplementary Table S1). These 10 proteins were not considered in further analysis. Then, a cross-comparison was performed to identify common proteins in the SF and RF groups (Figure 1C, yellow highlight in Supplementary Table S1). One increased and 36 decreased proteins were identified in both cryopreservation groups. One protein (Epididymal sperm-binding protein 1, ELSPBP1) was described as increased in the RF group and as decreased in the SF group.

### Enrichment Analyses of Differentially Expressed Proteins in Slow and Rapid Freezing Groups

DEPs in the SF group were significantly enriched with GO terms related to translation, protein targeting, viral infection, antigen processing, and metabolic processes (Figure 1D, Supplementary Table S2). On the other hand, in the RF group, DEPs were overrepresented with GO terms associated with exocytosis, proteasome, cell differentiation, cell cycle, metabolic processes, signaling pathways, protein processing and trafficking, translation, and mRNA processing (Figure 1E, Supplementary Table S2). For the RF group, biological processes and cellular compartment terms related to spermatozoon were also identified: “binding of sperm to zona pellucida”, “sperm-egg recognition”, “sperm capacitation”, “acrosomal vesicle” (also identified in the slow freezing group), “sperm flagellum”, “sperm fibrous sheath”, “acrosomal membrane”, “sperm principal piece” and “sperm connecting piece” (Figure 1E **,** Supplementary Table S2). The KEGG pathways enrichment analyses revealed that proteins from the SF group were associated with the ribosome, lysosome, and metabolic pathways, whereas proteins from RF were related to the proteasome, protein processing in the endoplasmic reticulum, signaling and metabolic pathways **(** Figures 1D,E **,** Supplementary Table S2).

The GO and KEGG terms of both groups were cross-compared to find common terms among them (highlight in blue in Supplementary Table S2). Eight common GO terms (“nuclear-transcribed mRNA catabolic process, nonsense-mediated decay”, “translational initiation”, “protein targeting to ER”, “SPR-dependent cotranslational protein targeting to membrane”, “azurophil granule lumen”, “acrosomal vesicle”, “azurophil granule” and “small ribosomal subunit”) and three common KEGG terms (“Glycolysis/Gluconeogenesis”, “Pyruvate metabolism” and “Cysteine and methionine metabolism”) were found. Those common terms were specifically related to the decreased proteins in both freezing groups.

### Proteasomal Proteins, Ribosomal Proteins, and Arylsulfatase a Are the Hubs in Integrative Networks

**FIGURE 2:**
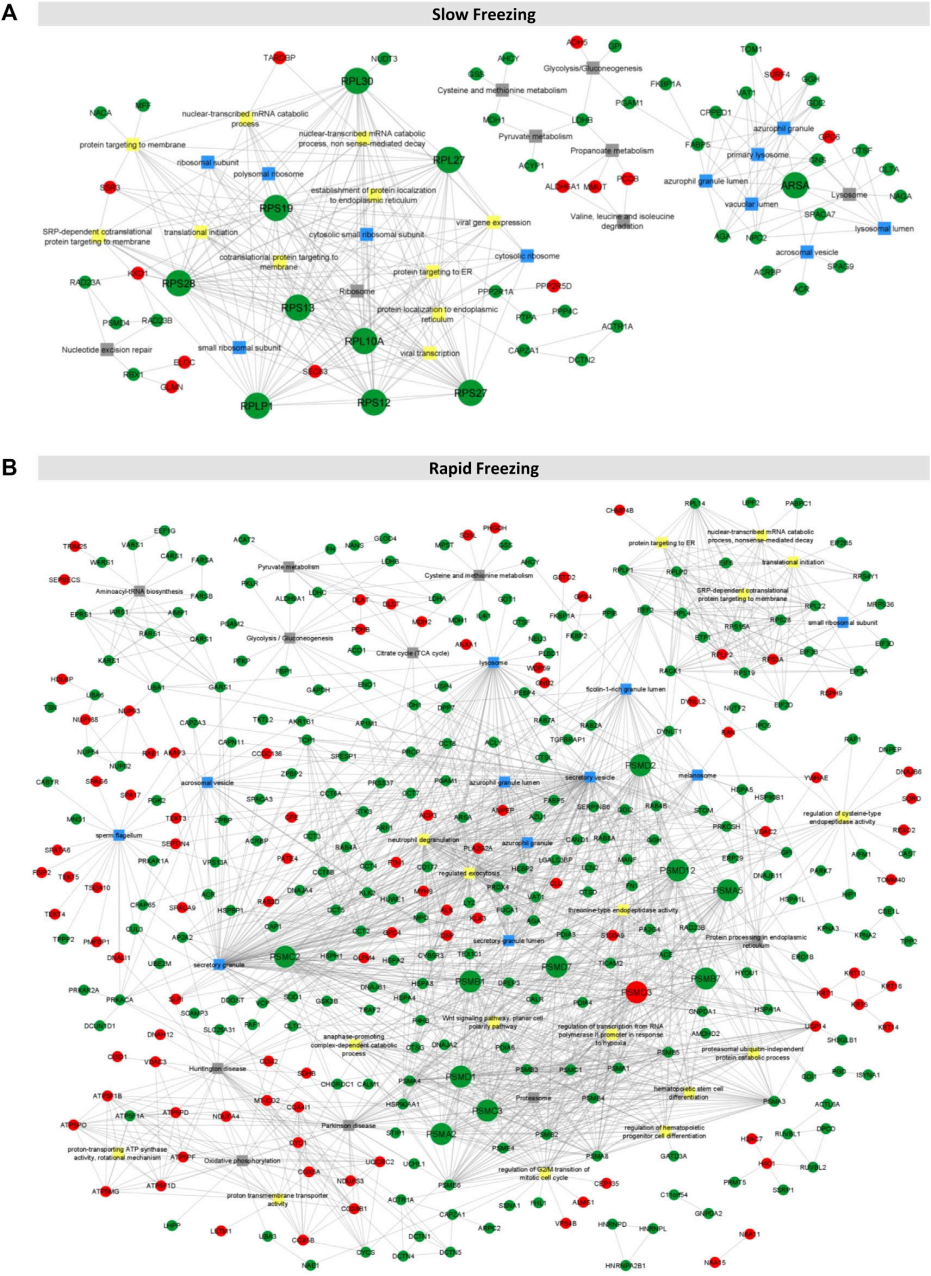
Integrative networks for rapid and slow freezing groups. **(A, B)** The integrative networks constructed for slow and rapid freezing groups using information from enrichment analyses (top 10 biological processes, cellular compartment, and KEGG terms as well as common terms to both groups and sperm-related terms) and PPIs data. Only connected proteins were represented (N = 60 and N = 344 for slow and rapid freezing, respectively). Each node corresponds to a DEP (represented by the gene name), being the red nodes increased proteins and the green nodes decreased proteins. The squares represent GO or KEGG terms (yellow squares for biological processes, blue squares for the cellular compartment, and grey squares for KEGG pathways). The nodes with bigger sizes represent the proteins with higher degree.

An integrative network for each freezing group was constructed using PPI and enrichment analysis data (Figure 2). In the String database, 54 and 607 PPIs were collected for the slow and rapid freezing groups, respectively. Noteworthy, for both groups, only PPIs with a high confidence score were selected to ensure highly probable interactions among the proteins in the cell. Based on node degree, a measure that indicates the number of connections of each node, top 10 hubs in each network was selected (represented with bigger circles in Figure 2 and Table 1). Most of the hubs in the SF network were cytoplasmic ribosomal proteins (Table 1). On the other hand, 11 hubs were identified in the RF (PSMC2, PSMC3, PSMD12, PSMD7 had the same node degree) and all hubs were proteasomal proteins (Table 1). Among SF hubs, the RPS28, RPS19, RPLP1, and ARSA proteins were also identified as decreased proteins in the RF group. None of the hubs has been described in the DISGENET database. ARSA was previously reported as decreased in poor quality sperm samples and male infertility-related conditions (Table 1). An additional search was performed in available proteomic studies carried out on human sperm (control condition vs male infertility/poor-sperm quality) to observe if any of the hubs had previously been identified as differentially expressed.

**TABLE 1:** Protein hubs identified in each integrative protein-protein interaction network: Slow Freezing (SF) and Rapid Freezing (RF). Node degree (computed in the respective network) and expression level (up or down) for each protein are indicated. Previous studies concerning expression levels of these proteins in conditions associated with poor-sperm quality are also indicated (studies were performed in human spermatozoa, except those where the specie is indicated). ZP, Zona Pellucida; N/A, not applicable.

UniProtKB	Gene name	Protein name	Degree	SF	RF	Previously described (level)
P25787	PSMA2	Proteasome subunit alpha type-2	38	N/A	↓	Asthenozoospermia (↑) Nowicka-Bauer et al. (2018)
P28066	PSMA5	Proteasome subunit alpha type-5	38	N/A	↓	
P20618	PSMB1	Proteasome subunit beta type-1	38	N/A	↓	
Q99436	PSMB7	Proteasome subunit beta type-7	38	N/A	↓	
Q99460	PSMD1	26S proteasome non-ATPase regulatory subunit 1	37	N/A	↓	
Q13200	PSMD2	26S proteasome non-ATPase regulatory subunit 2	37	N/A	↓	ZP Binding Failure (↑) Liu et al. (2018)
O43242	PSMD3	26S proteasome non-ATPase regulatory subunit 3	36	N/A	↑	Asthenozoospermia (↓) Liu et al. (2018)
						Globozoospermia (↑) Guo et al. (2019a)
P35998	PSMC2	26S proteasome regulatory subunit 7	35	N/A	↓	
P17980	PSMC3	26S proteasome regulatory subunit 6A	35	N/A	↓	Severe Asthenozoospermia (↑) Moscatelli et al. (2019)
O00232	PSMD12	26S proteasome non-ATPase regulatory subunit 12	35	N/A	↓	
P51665	PSMD7	26S proteasome non-ATPase regulatory subunit 7	35	N/A	↓	
P62857	RPS28	40S ribosomal protein S28	26	↓	↓	
P39019	RPS19	40S ribosomal protein S19	25	↓	↓	
P42677	RPS27	40S ribosomal protein S27	24	↓	N/A	
P62277	RPS13	40S ribosomal protein S13	24	↓	N/A	Asthenozoospermia (↓) Liu et al. (2015)
P25398	RPS12	40S ribosomal protein S12	24	↓	N/A	Asthenozoospermia (↑) Guo et al. (2019b)
P62888	RPL30	60S ribosomal protein L30	24	↓	N/A	Globozoospermia (↑) Guo et al. (2019a)
P62906	RPL10A	60S ribosomal protein L10a	23	↓	N/A	Globozoospermia (↑) Guo et al. (2019a)
P05386	RPLP1	60S acidic ribosomal protein P1	22	↓	↓	Globozoospermia (↑) Guo et al. (2019a)
P61353	RPL27	60S ribosomal protein L27	22	↓	N/A	
P15289	ARSA	Arylsulfatase A	7	↓	↓	Oxidative stress (↓) Bromfield et al. (2015)
						Globozoospermia (↓) Guo et al. (2019a)
						Asthenozoospermia (↓) Guo et al. (2019b)
						Low motility (↓) Amaral et al. (2014)
						Poor freezability boar sperm (↓) Guimarães et al. (2017)
						Defective bovine spermatozoa (↓) Kelsey et al. (2020)
						Impairment of mouse oocyte fertilization—mouse (↓) Caroselli et al. (2020)

### ARSA Levels Were Decreased After Slow and Rapid Freezing Cryopreservation

**FIGURE 3:**
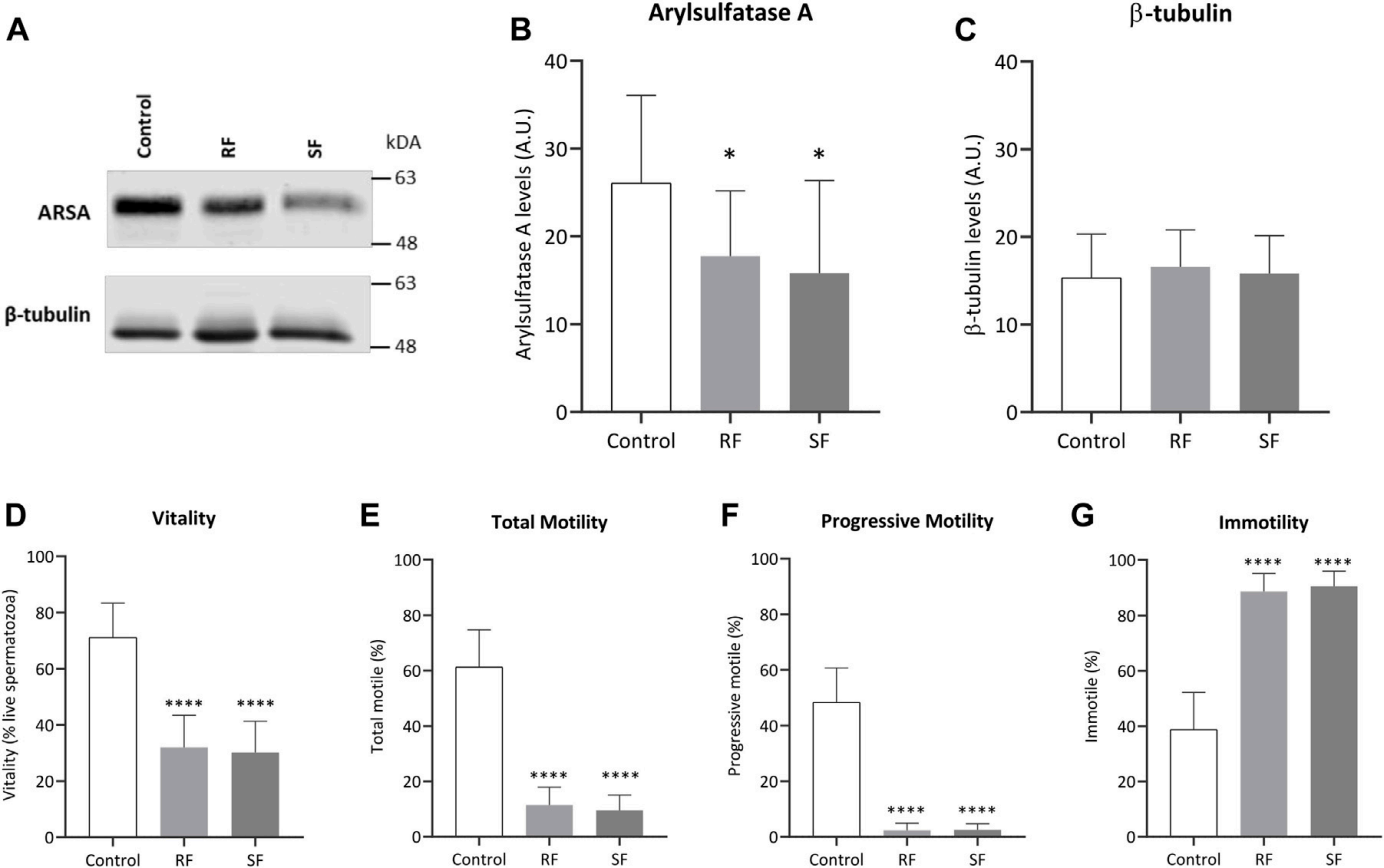
Impact of cryopreservation on ARSA levels and sperm parameters. **(A)** Representative image of ARSA (62 kDa) and *β*-tubulin (50 kDa) levels in ejaculated human spermatozoa, from normozoospermic men (*n* = 15), before and after rapid and slow freezing. Whole-cell lysates were separated by SDS-PAGE (**B,C**) Quantification of ARSA and *β*-tubulin levels, respectively (*n* = 15). For each condition, the band intensity was normalized to Ponceau staining. **(D)** Sperm viability was assessed through the eosin-nigrosine staining. The impact of the freezing method was also observed in sperm **(E)** total motility, **(F)** progressive motility, and **(G)** immotility. In all graphs, bars represent the mean values, and the error bars correspond to the standard deviation. Statistically significant findings compared with control are indicated with a *p < 0.05 or ****p < 0.0001. RF, rapid freezing; SF, slow freezing.

Among the hubs described in the prior section, ARSA was chosen for experimental investigation since it was decreased in both freezing groups and low ARSA levels have been consistently associated with poor sperm quality and male infertility-related conditions (Table 1). To assess the ARSA levels in freeze-thawed spermatozoa, sperm protein extracts were analyzed by western blot. The ARSA levels were significantly reduced after RF (∼31.97%) and SF (∼39.28%) samples (Figures 3A,B). The post hoc power analysis revealed that differences between control and RF and SF groups yielded a statistical power of 75,32% and 74,63%, respectively. No significant alterations were observed in *β*-tubulin levels (Figures 3A,C). Cell viability and sperm motility were also evaluated. As expected, cell viability was significantly decreased in freeze-thawed spermatozoa (Figure 3D). Total and progressive motilities were significantly decreased in RF and SF (Figures 3E,F, respectively) and the percentage of immotile sperm was increased (Figure 3G).

## Discussion

Cryopreservation induces significant cellular changes in spermatozoon that are extensively described though poorly understood from a molecular point of view (Hezavehei et al., 2018; Rienzi et al., 2018; Kumar et al., 2019). In recent years, proteomic analysis has become an important tool to identify DEPs allowing the molecular understanding of certain physiological and pathological contexts (Li et al., 2017; Agarwal et al., 2020). Still, one of the major limitations of examining single proteomic studies is to draw robust conclusions concerning potential markers and altered pathways in a specific condition. To date, the present study is the first to merge all the available proteomic data from human cryopreserved sperm to unveil key DEPs that can be potential cryopreservation markers or modulation targets.

From the included proteomic studies (Bogle et al., 2017; Fu et al., 2019; Li et al., 2019), decreased and increased DEPs were collected for the two conventional freezing techniques used in sperm cryopreservation: slow freezing (SF) and rapid freezing (RF). Decreased protein levels are easily explained by the cryopreservation process, which leads to protein degradation, plasmatic membrane damage, and osmotic stress induced by the cryoprotectant. Antagonistically, the underlying mechanisms for increased proteins levels are less clear. First, the protein increased levels are not induced by the cryoprotectant used, since included studies only used egg-yolk-free mediums (Bogle et al., 2017; Fu et al., 2019; Li et al., 2019). Wang and colleagues (Wang et al., 2014) conjectured that post-translational modifications (PTMs), such as protein phosphorylation, could be the reason behind the protein increment. In fact, cryopreservation appears to induce a set of differential protein-tyrosine phosphorylation patterns (Naresh and Atreja, 2015). Nonetheless, despite PTMs being identified as perturbations in protein abundance (Larsen et al., 2006), shotgun proteomics (the same approach used in the included studies) has various limitations in PTMs analysis and enrichment protocols are necessary to a proper PTMs study (Agarwal et al., 2020; Virág et al., 2020). Thus, further investigation should be performed to understand why some proteins are increased after cryopreservation.

Integrative networks using GO and KEGG pathways enrichment analyses, and PPI data allowed the identification of relevant proteins in each freezing group network. In the SF network, nine of the 10 hubs were cytoplasmic ribosomal proteins (RPS28, RPS19, RPS27, RPS13, RPS12, RPL30, RPL10A, RPLP1, and RPL27). Despite mammalian spermatozoa has been considered as transcriptionally and translationally inherent, some studies reported the existence of *de novo* protein synthesis under capacitation conditions (Gur and Breitbart, 2006, 2008; Li et al., 2010; Rajamanickam et al., 2017; Zhu et al., 2019). The protein translation occurrence appears to be important for the replacement of some degraded proteins during capacitation events and its impairment was associated with a reduction in motility, actin polymerization, acrosomal reaction, and *in vitro* fertilization (Gur and Breitbart, 2006). In the light of current knowledge, no available studies are referring neither the occurrence of translation activity in freeze-thawed spermatozoa nor the association between low levels of ribosomal proteins with poor-sperm quality. Nevertheless, the GO and KEGG pathways analyses revealed that decreased proteins from the SF group were strongly associated with translation-related terms, suggesting that translation pathways were negatively affected by cryopreservation. Further approaches should be performed to assess the translation activity in freeze-thawed sperm and the impact of translation impairment after cryopreservation.

By contrast, in the RF network, all hubs were proteasomal proteins (PSMA2, PSMA5, PSMB1, PSMB7, PSMD1, PSMD2, PSMD3, PSMC2, PSMC3, PSMD12, and PSMD7), which are components of the 26S proteasome. The 26S proteasome is a multi-catalytic protease with a high affinity for ubiquitinated proteins and has a crucial role in protein degradation and turnover in all living organisms (Livneh et al., 2016). In mammalian spermatozoa, 26S proteasome has been implicated in sperm capacitation and fertilization events. Recent evidence showed that 26S proteasome activity is increased during the early onset of capacitation, through activating phosphorylation by protein kinase A (Kerns et al., 2016). Sperm active proteasome participates in protein degradation and modification “helping” the plasma membrane and acrosome remodulation, acrosomal exocytosis, and sperm-zona pellucida (ZP) interaction (Kerns et al., 2016). If proteasomal proteins are decreased, the 26S proteasome pathway could be affected leading to capacitation failure and consequently fertilization inability of freeze-thawed spermatozoa. In fact, decreased proteasomal activity was already associated with poor-sperm quality (Rawe et al., 2008; Rosales et al., 2011). Additionally, the enrichment analyses revealed a significant association between decreased DEPs from the RF group and proteasome pathway**,** corroborating this hypothesis. Thus, this study presents the first evidence that the proteasomal pathway could be a good target for future studies in sperm cryopreservation.

Among the hubs identified, arylsulfatase A (ARSA) had decreased levels in both freezing groups and thus it was selected for subsequent investigation after cryopreservation. ARSA is a lysosomal enzyme present in sperm membrane that interacts, before gametes fusion, with sulfated sugar residues of ZP-glycoproteins and, consequently, contributes to sperm binding and penetration of the ZP (Xu et al., 2012a). Together with hyaluronidase PH-20 (SPAM1) and Heat shock-related 70 kDa protein 2 (HSPA2), they form a protein-membrane complex being only expressed on sperm surface after capacitation (Redgrove et al., 2012, 2013; Gómez-Torres et al., 2021). ARSA was identified in proteomic studies of cryopreserved human sperm (Fu et al., 2019; Li et al., 2019). Still, this is the first study to highlight ARSA potential as a marker to assess sperm quality after cryopreservation and to perform further experimental validation. ARSA levels were analyzed in freeze-thawed human sperm samples, being significantly decreased after both SF and RF cryopreservation. This observation suggests that cryopreservation methods induce ARSA degradation. As expected, both methods also induced a drastic decrease in sperm viability and motility. Low ARSA levels were previously reported after cryopreservation in boar sperm with poor freezability and defective bovine sperm (Guimarães et al., 2017; Kelsey et al., 2020). Besides cryopreservation, ARSA reduced levels were observed in sperm exposed to oxidative stress inducers (Bromfield et al., 2015), sperm with low motility (Amaral et al., 2014), and globozoospermia (Guo et al., 2019a) and asthenozoospermia (Guo et al., 2019b) conditions. Together, these findings suggest ARSA as a potential good biomarker to access sperm quality after cryopreservation and to distinguish between good- and poor-sperm. Curiously, the oxidative stress effects in ARSA expression levels appeared to be minimized in sperm cells treated with penicillamine (Bromfield et al., 2015). Caroselli and colleagues showed that ARSA could be reversibly inhibited in mouse sperm and its inhibition significantly impaired the *in vitro* oocyte fertilization (Caroselli et al., 2020). Therefore, considering the ARSA key role in sperm-oocyte interaction (Xu H. et al., 2012), future studies should be performed to prevent ARSA degradation and increase the success of cryopreservation protocols.

The present work presents three main limitations. Considering the importance of cryopreservation protocols in reproductive medicine, there is a very limited number of proteomic studies on human sperm available. Second, there is limited knowledge in the databases for a cell type as specialized as the sperm cell. Most signaling pathways have been thoroughly investigated in somatic cells. However, they are poorly characterized in male germ cells. This negatively affects the bioinformatic analyses performed in this study. The last limitation concerns the small number of samples included in experimental validation. Despite the high statistical power yielded, the validation can be strengthened by increasing the number of men recruited.

To summarize, the integration of the available proteomic data, using a bioinformatic workflow, proved to be very useful to identify a set of key proteins that are differentially expressed in freeze-thawed human spermatozoa and to highlight ARSA for further experimental validation. For the first time, ARSA levels were assessed in freeze-thawed human sperm, being significantly decreased in both freezing groups. Thus, ARSA has the potential to be a good marker to evaluate sperm quality after cryopreservation. On the other hand, the enrichment analysis also allowed to recognize, for the first time, two altered pathways (proteasomal and translation pathways) on cryopreserved human sperm that could be investigated in future research.

## Data Availability

The original contributions presented in the study are included in the article/Supplementary Material, further inquiries can be directed to the corresponding author.

## Abbreviations

ARSA, Arylsulfatase A; DEPs, Differentially Expressed Proteins; GO, Gene Ontology; PPI, Protein-Protein Interaction; RF, Rapid Freezing; SF, Slow Freezing; ZP, Zona-Pellucida.
