# Supplementary material for: Bioinformatic Approach to Unveil Key Differentially Expressed Proteins in Human Sperm After Slow and Rapid Cryopreservation
Source: Front Cell Dev Biol. 2022 Jan 25;9:759354. doi: 10.3389/fcell.2021.759354 (PMC8821918; doi:10.3389/fcell.2021.759354)
Supplement: Supplementary file 2 [file DataSheet1.pdf]

Supplementary Material

1 Supplementary Tables

Table S1 - Differentially expressed proteins (DEPs) in spermatozoa cryopreserved through slow and rapid freezing methods (SF and RF, respectively). All accession numbers identified in selected studies were collected and mapped in the UniProt database. All proteins were annotated with UniProtKB/SwissProt number to avoid redundancy. Proteins with a reviewed status were also identified in the UniProt database. A cross-comparison was performed to identify proteins previous reported in sperm proteomic studies (Santiago et al., 2019). Proteins yellow highlighted were identified in both freezing groups. Proteins blue highlighted were described as increased and described in different studies in RF group.

Table S2 – Enrichment analyses of differentially expressed proteins (DEPs) in slow and rapid freezing groups (SF and RF, respectively). For each group, Gene Ontology and KEGG pathway analyses were performed to identify significant terms associated with the DEPs. Terms blue highlighted were identified in both freezing groups. BP, Biological Process; CC, Cellular compartment; MF, Molecular Function.
